# Organelle_PBA, a pipeline for assembling chloroplast and mitochondrial genomes from PacBio DNA sequencing data

**DOI:** 10.1186/s12864-016-3412-9

**Published:** 2017-01-07

**Authors:** Aboozar Soorni, David Haak, David Zaitlin, Aureliano Bombarely

**Affiliations:** 1 Department of Horticulture, Virginia Polytechnic Institute and State University, Blacksburg, VA 24061 USA; 2 Department of Horticulture, Faculty of Horticultural Sciences and Plant Protection, University of Tehran, Karaj, 31587 Iran; 3 Department of Plant Pathology, Physiology and Weed Science, Virginia Polytechnic Institute and State University, Blacksburg, VA 24061 USA; 4 Kentucky Tobacco Research and Development Center (KTRDC), University of Kentucky, Lexington, KY 40546 USA

**Keywords:** Chloroplast, Mitochondria, PacBio, Organelle Genome Assembly

## Abstract

**Background:**

The development of long-read sequencing technologies, such as single-molecule real-time (SMRT) sequencing by PacBio, has produced a revolution in the sequencing of small genomes. Sequencing organelle genomes using PacBio long-read data is a cost effective, straightforward approach. Nevertheless, the availability of simple-to-use software to perform the assembly from raw reads is limited at present.

**Results:**

We present Organelle-PBA, a Perl program designed specifically for the assembly of chloroplast and mitochondrial genomes. For chloroplast genomes, the program selects the chloroplast reads from a whole genome sequencing pool, maps the reads to a reference sequence from a closely related species, and then performs read correction and *de novo* assembly using Sprai. Organelle-PBA completes the assembly process with the additional step of scaffolding by SSPACE-LongRead. The program then detects the chloroplast inverted repeats and reassembles and re-orients the assembly based on the organelle origin of the reference. We have evaluated the performance of the software using PacBio reads from different species, read coverage, and reference genomes. Finally, we present the assembly of two novel chloroplast genomes from the species *Picea glauca* (Pinaceae) and *Sinningia speciosa* (Gesneriaceae).

**Conclusion:**

Organelle-PBA is an easy-to-use Perl-based software pipeline that was written specifically to assemble mitochondrial and chloroplast genomes from whole genome PacBio reads. The program is available at https://github.com/aubombarely/Organelle_PBA.

**Electronic supplementary material:**

The online version of this article (doi:10.1186/s12864-016-3412-9) contains supplementary material, which is available to authorized users.

## Background

Single Molecule Real Time (SMRT) sequencing technology developed by Pacific Biosciences (PacBio), can produce millions of long reads (1 Kb or longer, with a current average of 12Kb) per run. SMRT sequencing is based on single molecule real-time imaging of the incorporation of fluorescently tagged nucleotides to a DNA template molecule [1]. This technology has been successfully applied to a wide range of experiments and species such as the sequencing of DNA amplicons [2] and transcriptomes [3]. Nevertheless the most popular application is whole genome sequencing. It has been used for the sequencing of bacterial genomes such as the plant pathogen *Xanthomonas oryzae* [4]. PacBio reads have also been used for the sequencing of complex plant nuclear genomes, such as that of the Adzuki bean, *Vigna angularis* [5], demonstrating the advantage of this technology for resolving repetitive regions during sequence assembly.

Sequence assembly is the process whereby one or more consensus sequences are reconstructed from hundreds to billions of individual DNA sequence reads. Although there are dozens of programs to produce consensus sequences, they can be classified into two groups based on the algorithm they use: Overlap–Layout–Consensus (OLC) and De Bruijn Graph (DBG). OLC algorithms are best suited for low coverage long read approaches. The most popular PacBio assemblers such as HGAP [6] and Falcon (https://github.com/PacificBiosciences/falcon) utilize OLC algorithms. A popular OLC-based program, the Celera Assembler (CA; [7]), has been updated to assemble PacBio reads [8] (this program, called PBcR, is being replaced by Canu [http://canu.readthedocs.org/] and is no longer maintained). Another option is the use of Sprai [9], a pipeline that employs the CA assembly algorithm. This pipeline pre-selects the best 20X coverage reads using BLAST searches [10], then corrects and assembles them using CA. PacBio sequencing and OLC assemblers have been successfully applied to the sequencing of yeast mitochondrial genomes [11] and chloroplast genomes such as those of *Potentilla micrantha* [12], *Nelumbo nucifera* [13], and sugar beet (*Beta vulgaris*) [14].

Mitochondrial and chloroplast DNA markers are the bridge between population genetics and systematics, primarily because they are maternally inherited and do not recombine; thus they can facilitate the reconstruction of maternal lineages [15]. Mitochondrial genomes vary in size depending on the eukaryotic lineage. For animals, the lengths range from 28,757 bp (*Breviceps adspersus*) [16] to 13,424 bp (*Didemnum vexillum*) [17] with the average size being 16,800 bp. Conversely, mitochondrial genomes of plants and fungi can vary by almost three orders of magnitude, ranging from 15,758 bp (*Chlamydomonas reinhardtii*) [18] to 11.3 Mb (*Silene conica*) [19]. Chloroplast genomes, on the other hand, are typically much more conserved in their size and structure, ranging from 11,348 bp (*Pilostyles aethopica*) [20] to 521,168 bp (*Floydiella terrestris*) [21], with an average size of ca. 148,000 bp. At present, >10,000 mitochondrial genomes have been sequenced, while comparatively fewer (~990) chloroplast genomes have been sequenced (http://www.ncbi.nlm.nih.gov/genome).

The application of PacBio long-read DNA sequencing technology to organelle genome sequencing will duplicate the numbers given above in the next 2 years. As previously described, there are several tools designed to assemble PacBio reads (e.g. HGAP, Falcon, Canu, and Sprai); however, no single tool is available to assemble organelle genomes using total DNA sequencing reads derived from the PacBio platform. We have developed a new Perl-based tool, Organelle_PBA, designed expressly to reconstruct whole organelle genomes from PacBio data. First, the program selects the specific organelle reads by mapping raw reads to a reference organelle genome. Then, it produces a de-novo assembly using Sprai, a new re-scaffolding program, and removes the redundancy produced by the circular organization of these genomes. Organelle_PBA also resolves the inverted repeats found in chloroplast genomes. The tool is available at https://github.com/aubombarely/Organelle_PBA.

### Material and methods

PacBio reads from *Arabidopsis thaliana* (SRR1284093, SRR1284094, SRR1284095, SRR1284703, SRR1284704), *Mus musculus* (ERR731675) and *Picea glauca* (SRR2148116) were downloaded from the SRA repository using the Prefech program from the SRA Toolkit. The SRA file format was then converted to Fastq format using the Fastq-dump program in the SRA Toolkit.

*Sinningia speciosa* PacBio reads were obtained by *de novo* PacBio DNA sequencing. Briefly, the *S. speciosa* variety ‘Avenida Niemeyer’ [22] was grown under fluorescent lighting at room temperature (~23 °C). DNA was extracted from young flower buds using the Qiagen DNEasy® extraction kit. DNA was quantified using a Nanodrop® ND-1000 spectrophotometer, and its integrity was evaluated by agarose gel electrophoresis. DNA was sent to the Duke Center for Genomics and Computational Biology facility, where a SMRTBell™ long insert PacBio library (15–20Kb fragments) was prepared and then sequenced using a PacBio RSII system (P6-C4 Chemistry). PacBio reads were used directly in Organelle_PBA without any extra processing.

Organelle reference genomes were downloaded from the NCBI nucleotide database. The downloaded references were *M. musculus*, NC_005089.1; *Mus carolis*, NC_025268.; *Rattus norvegicus*, NC_001665.2; *Marmota himalayana*, NC_018367.1; *A. thaliana*, NC_000932.1 and NC_001284.2; *Brassica napus*, NC_016734.1; *Vitis vinifera*, NC_007957.1; *P. abies* NC_021456.1 and *Boea hygrometrica* NC_016468.1*.*

For organelle genome coverage evaluation, the PacBio reads were mapped using BlasR [23] with the sam output format parameter. The result was piped into SAMtools for filtering of the unmapped reads [24]. Coverage was calculated using BEDtools [25], and variants, SNPs, and InDels were called using FreeBayes [26].

## Implementation

Organelle_PBA is a program written in Perl constructed as a single file script. It uses the following Perl 5.18 modules: Getopts::Std (core), File::Spec (core), File::Basename (core), File::Copy (core), File::Path (core), IPC::Cmd (core), Math::BigFloat (core), Bio::SeqIO (bioperl) and Bio::Tools::Run::StandAloneBlastPlus (bioperl). Additionally, it uses the following programs: BlasR [23], SAMtools [24], Blast [10], SSPACE-LongRead [27], Sprai [9] and BEDtools [25]. The executable files are passed to the script as environmental variables if they are not accessible from the PATH. Upon initial run, the script checks that all of the executable files are available prior to initializing any processes. The subprograms are run using the IPC::Cmd Perl module.

The program is divided into the following steps (Additional file 1: Figure S1):

0. Argument check and analysis of the input stats such as organelle reference size and number of reads.
1. Mapping of the PacBio reads to the organelle reference genome using BlasR.
2. Parsing of the BlasR results and selection of the PacBio reads that map to the organelle reference. The percentage of the read length aligned to the reference can be used to filter these hits.
3. Read correction and assembly using Sprai using the reads selected in the previous step. Reads can be filtered by length before the assembly using the Sprai arguments.
4. Assembly evaluation comparing the total assembly size and the longest contig size with the reference sequence size. If the longest contig is longer than the reference genome, the script moves to step 6 (circular assembly check), otherwise it continues to step 5.
5. If the longest contig size is smaller than the reference, Organelle_PBA interprets the assembly as being fragmented. It then runs BlasR and SSPACE-LongRead with the entire read dataset to find any reads that it could not select during the BlasR mapping (Step 1). After this, it evaluates the assembly again reporting the new sizes and then moving to step 6.
6. Circular assembly check by homology search (Blast) of the assembled sequence with itself. The program also checks for a possible origin based on the reference through a homology search (Blast). If it finds any of these, it will break the contig/scaffold, reorganizing the pieces to remove the redundancy from a circular assembly and orient the assembly based on the reference genome sequence.
7. Check the completeness of the assembly. Chloroplast genomes are composed of four parts: Long Single Copy (LSC) section, Short Single Copy (SSC), and two Inverted Repeats: IRa and IRb. The inverted repeats, IRa and IRb, are identical and sometimes are only partially assembled, so the assembly could appear to be complete with a size smaller than the reference. If this is the case, the program will move to step 8, if it is not, it will move to step 9.
8. Inverted repeat evaluation and resolution. Organelle_PBA will map all the reads back to the assembly using BlasR to calculate the coverage for each part of the assembly. Inverted repeats appear with twice the coverage of the non-repeated region as a result of the multiple mapping sites reported by BlasR (see Results). Additionally, the program will break these regions looking for sequence homology using Blast to analyze if they present a certain level of homology reported by BlastN. If the script finds it, it will remove the redundancy and rebuild the assembly using SSPACE-LongRead.
9. Final assembly analysis and assembly statistics report printing.

## Results

### *Mus musculus* mitochondrial genome assembly

**Fig. 1:**
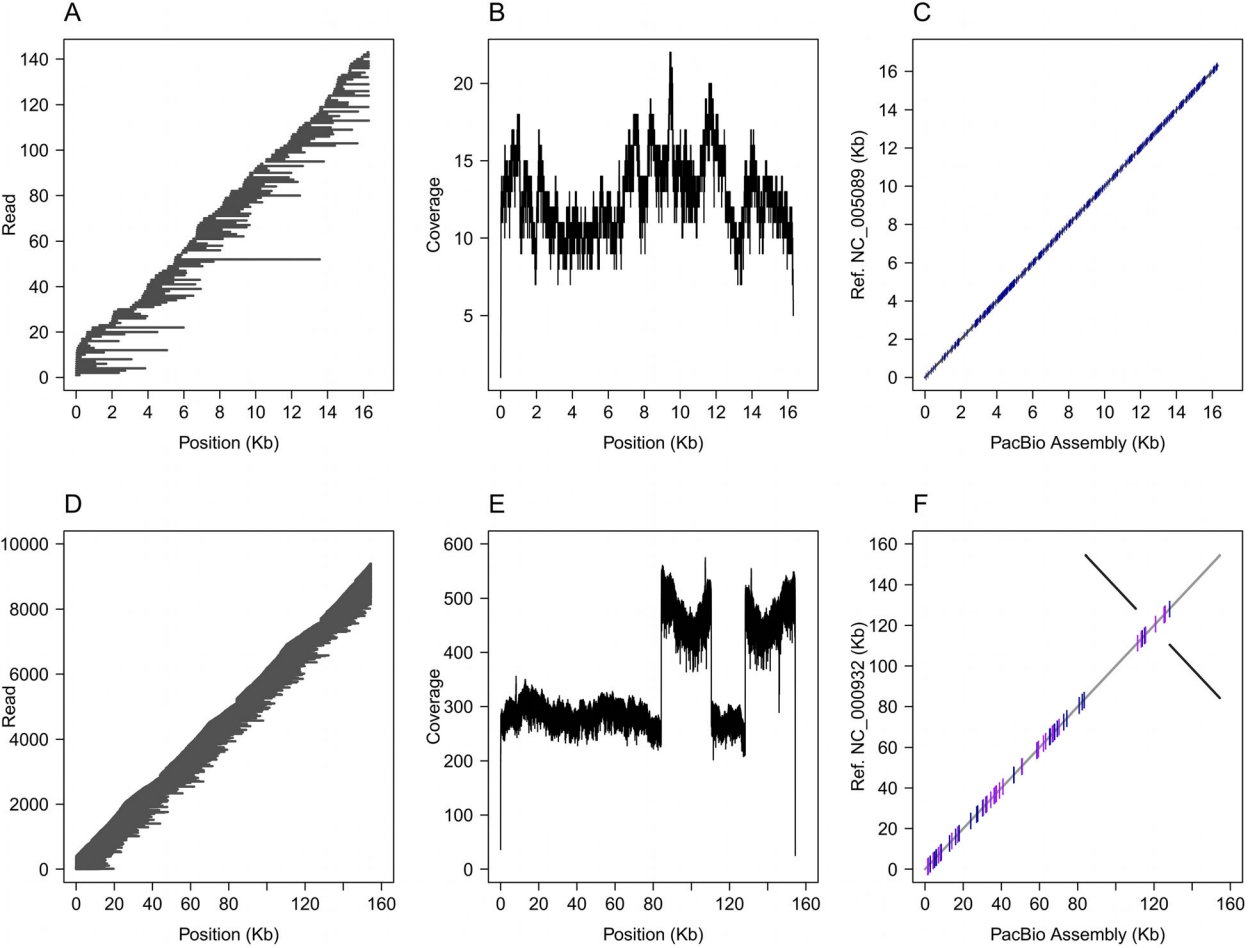
**a**- Remapping of *Mus musculus* PacBio DNA sequencing reads to a mitochondrial genome reference assembly. Each of the reads is represented by a darkgray line marking its position. **b**- Coverage for the PacBio read remapping for *M. musculus*. **c**- Alignment between the *M. musculus* reference mitochondrial genome (NC_005089.1) and the *M. musculus* assembly performed by Organelle_PBA. SNPs are represented by small horizontal blue lines. **d**- Remapping of *Arabidopsis thaliana* PacBio sequencing reads to a reference chloroplast genome assembly. Each of the reads is represented by a darkgray line marking its position. **e**- Coverage for the PacBio read remapping for *A. thaliana*. The inverted repeats are indicated by ~2X coverage relative to the LSC and SSC regions. **f** - Alignment between the *A. thaliana* reference chloroplast genome (NC_000932.1) and the *A. thaliana* assembly performed by Organelle_PBA. SNPs and Indels are represented by small horizontal blue and purple lines respectively. Reversed alignments are represented by darkgray lines

Sets of 50,000, 100,000, and 163,477 randomly selected PacBio reads from the house mouse, *Mus musculus* (SRA datatset: ERR731675), were used to test the mitochondrial genome assembly using different mitochondrial reference genomes; *M. musculus*, NC_005089.1 (same species); *Mus carolis*, NC_025268.1 (same genus); *Rattus norvegicus*, NC_001665.2 (same family - Muridae) and *Marmota himalayana*, NC_018367.1 (different family - Sciuridae). Results are summarized in the Table 1. The different read sets represented 26X, 42X, and 69X sequencing depth, respectively, for the *M. musculus* mitochondrial genome (~0.2% of the downloaded PacBio dataset). Organelle_PBA produced a complete *M. musculus* mitochondrial genome assembly for the 163,477 reads set (69X) with all of the reference sequences except for the *M. himalayana* mitochondrial genome. The average running time for this process was 117 s. The PacBio read remapping showed that the assembly was fully covered (from ~5X to ~25X) with no gaps (Fig. 1a and b). Additionally, a comparison of the assembled mitochondrial genome with the *M. musculus* mitochondrial reference (NC_005089.1) showed a perfect alignment with 395 SNPs distributed across the entire assembly (Fig. 1c).

**Table 1:** Summary of the *M. musculus* mitochondrial genome assembly

Input reads	Reference	Mapped reads	% Bases mapped	Estimated depth (x)	Assembly size (bp)	Organelle completed^a^
50,000	*Mus musculus*	39	0.22	26	2678	NO
100,000		83	0.18	42	12,377	NO
163,477		138	0.22	69	16,294	YES
50,000	*Mus caroli*	35	0.20	24	NA	NO
100,000		69	0.16	36	12,332	NO
163,477		110	0.15	56	16,299	YES
50,000	*Rattus norvegicus*	26	0.16	19	NA	NO
100,000		53	0.13	30	10,247	NO
163,477		86	0.12	44	16,292	YES
50,000	*Marmota himalayana*	10	0.07	8	NA	NO
100,000		17	0.05	11	6580	NO
163,477		31	0.04	17	7193	NO

^a^The mitochondria genome assembly was considered complete when the difference in size compared to the reference genome was <10 nucleotides

To compare the performance of this approach compared with a whole dataset assembly and posterior identification of the organelle genome, we performed a whole dataset assembly using Canu and Sprai using similar configuration parameters. Canu assembled the 163,477 reads in 3970 s producing 7 contigs with a L50 = 17,784 bp. We identified a 15,206 bp mitochondrial genome contig by BlastN homology search. Sprai assembled the same dataset in 3660 s producing 5 contigs with a L50 = 5858 bp. None of the Sprai contigs were identified as the mitochrondrial genome.

Based on these results, we can conclude that >50X sequencing depth and a reference genome sequence from the same taxonomic family is recommended for the assembly of a full mammalian mitochondrial genome.

### *Arabidopsis thaliana* chloroplast genome assembly

Sets of 5000, 10,000, 50,000, 100,000, and 163,448 randomly selected PacBio reads from *Arabidopsis thaliana* (SRA dataset: SRR1284093) were used to test the chloroplast genome assembly using different chloroplast references; *A. thaliana*, NC_000932.1 (same species); *Brassica napus*, NC_016734.1 (same family); and *Vitis vinifera*, NC_007957.1 (family Vitaceae). Results are summarized in Table 2. The different read sets represented 23X, 50X, 244X, 470X, and 771X sequencing depth for the *A. thaliana* chloroplast genome, respectively (~23% of the downloaded PacBio dataset). The running time for the whole set (163,448 reads) was 5234 s. The assembly was fully covered with no gaps (Fig. 1d), and showed coverage ranging from ~50X to ~600X. The inverted repeats showed twice the average coverage of the LSC and SSC regions (Fig. 1e). The comparison with the *A. thaliana* reference (NC_000932.1) showed 37 SNPs, 53 insertions, and 73 deletions in the LSC and SSC regions (Fig. 1f).

**Table 2:** Summary of the *A. thaliana* chloroplast genome assembly

Input reads	Reference	Mapped reads	% Bases mapped	Estimated depth (x)	Assembly size (bp)	Organelle completed^a^
5000	*Arabidopsis thaliana*	287	23.06	23	42,978	NO
10,000		611	24.40	50	150,039	NO
50,000		3,013	23.79	244	154,472	YES
100,000		5,777	23.01	470	154,471	YES
163,448		9,409	23.15	771	154,474	YES
5000	*Brassica napus*	277	21.82	22	59,513	NO
10,000		591	23.08	48	153,132	NO
50,000		2923	23.08	239	154,474	YES
100,000		5565	22.13	457	154,481	YES
163,448		9102	22.43	755	154,473	YES
5000	*Vitis vinifera*	233	18.88	18	73,382	NO
10,000		507	20.62	41	151,984	NO
50,000		2516	20.67	204	154,469	YES
100,000		4807	20.04	393	154,477	YES
163,448		7855	20.28	649	154,472	YES

^a^The chloroplast genome assembly was considered complete when the difference in size compared to the reference genome was <10 nucleotides

Additionally we performed the whole dataset assembly and posterior chloroplast identification by BlastN sequence homology to compare with our approach where the read identification is performed before the assembly. Canu produced 31 contigs in 6040 s with a L50 of 32,701 bp. We identified three chloroplastic contigs with lengths of 117,666, 53,847, 30,575 bp respectively. Sprai produced 2 contigs in 8591 s. The longest sequence represented the complete chloroplast genome with a length of 163,611 bp including a redundant region of 9133 bp caused by the circularity of the chloroplast genome.

Based on these results, we conclude that it is necessary to have at least 200X sequencing depth to obtain a fully assembled chloroplast genome. Considering the taxonomic distance of the reference sequence, we can suggest that almost any chloroplast genome from a species in the same subclass should be usable in selecting the chloroplast reads. We did not test the use of chloroplast reference genomes from other subclasses (e.g. an asterid reference to assemble the rosid *A. thaliana* chloroplast genome) because there are enough reference genomes from the same subclass publically available. The use of a whole dataset assembly with Sprai and posterior identification delivered the complete chloroplast genome sequence, although this approach used 64% more time without counting a final result curation to remove the redundant region.

### *Arabidopsis thaliana* mitochondrial genome assembly

Sets of 5000, 10,000, 50,000, 100,000, 163,448, 490,143, and 817,099 randomly selected PacBio reads from *Arabidopsis thaliana* (SRA datasets: SRR1284093, SRR1284094, SRR1284095, SRR1284703 and SRR1284704) were used to test the mitochondrial genome assembly using the *A. thaliana* mitochondrial reference sequence (NC_001284.2). The results of the assemblies are summarized in Table 3. No complete mitochondrial genome assembly was obtained using this methodology. The mapping of the full *A. thaliana* dataset SRR1284093 (163,448 reads) to the *A. thaliana* mitochondrial genome NC_001284.2 selected 3046 reads (equivalent to 111X) but a close inspection showed an average coverage of 9X with 14,533 non-covered positions; thus, the selection of mapped reads for this dataset does not represent the full mitochondrial genome. Increasing the number of reads to 490,143 and 817,099 also increased the effective coverage to 33X and 64X, but there were still 8045 and 6305 non-covered positions, respectively, that produced incomplete assemblies.

**Table 3:** Summary of the *A. thaliana* mitochondrial genome assembly

Input reads	Reference	Mapped reads	% Bases mapped	Estimated depth (x)	Assembly size (bp)	Organelle completed^a^
5000	*Arabidopsis thaliana*	101	8.88	4	27,294	NO
10,000		215	8.80	8	57,303	NO
50,000		1,006	8.63	37	156,177	NO
100,000		1,861	7.92	68	152,405	NO
163,448		3,046	7.92	111	177,810	NO
490,143		11,080	8.31	434	136,334	NO
817,099		21,099	8.72	829	150,873	NO

^a^The mitochondrial genome assembly was considered complete when the size difference compared to the reference was <100 nucleotides

### Novel organelle genome assemblies

To test the ability of Organelle_PBA to assemble new plant organelle genomes, we performed chloroplast genome assemblies for two species *Picea glauca* (white spruce) and *Sinningia speciosa* (a wild form of the cultivated florist’s gloxinia). For *P. glauca*, a read dataset with 563,675 reads was downloaded from the SRA repository (SRR2148116). For *Sinninigia speciosa*, a set of 923,290 reads was selected from the *S. speciosa* whole genome sequencing project (manuscript in preparation). There is no chloroplast genome assembly available for *P. glauca*, but there is a chloroplast genome reference from a species in the same genus, *P. abies* (NC_021456.1). The *P. glauca* chloroplast assembly produced a 123,423 bp contig with 237 nucleotide insertions, 302 nucleotide deletions, and 416 SNPs compared with the *P. abies* chloroplast genome. For the *S. speciosa* chloroplast genome assembly we used the *Boea hygrometrica* chloroplast genome (same family as *S. speciosa*, but different subfamily). The *S. speciosa* assembly produced a 153,428 bp contig with 2212 nucleotide insertions, 1766 nucleotide deletions, and 6783 SNPs compared with the *B. hygrometrica* chloroplast genome sequence.

## Discussion

Organelle_PBA is a script designed to assemble organelle genomes from long-read whole genome sequencing data by selecting the organelle reads and then mapping them to a closely related reference sequence. Animal mitochondria and plant chloroplast genomes are generally highly conserved across different lineages, so the use of a reference sequence from the same family is usually enough to select reads and then perform a focused assembly, thereby reducing the use of computational resources. Our results indicate that coverage between 50 and 200X is usually enough to obtain a full organelle genome assembly. Our analysis shows that an average whole genome sequencing project contains ~0.2% of animal mitochondrial DNA and ~20% of plant chloroplast DNA, which, in most cases, is enough to reach >50X organelle genome coverage. Mapping of these reads shows that they are equally distributed across the organelle genome such that the mapping strategy employed effectively captures the full representation of the organelle genome. Nevertheless, the assembly of plant mitochondrial genomes can be difficult because they are more variable in size and genomic composition, and they are usually poorly represented in whole genome sequencing datasets. The assembly of the *A. thaliana* mitochondrial genome was incomplete, likely due to incomplete mapping to the reference genome. Additionally, Sprai selects only the best 20X coverage of the PacBio reads to perform the assembly, so unequal mapping could introduce bias into the Sprai read selection. We also found, through coverage analysis, two peaks of high coverage (>1000X) that probably represent highly repetitive regions, although a more detailed analysis needs to be performed to verify this hypothesis.

Finally, even if assembly of an organelle genome is not the final goal of a whole genome sequencing project, there are some advantages to assembling the organelle genome prior to launching the nuclear genome assembly: 1) It can facilitate assembly of the nuclear genome by reducing the amount of data used in the whole genome assembly that in some cases can reach 25% or more (e.g. chloroplast DNA in plants); 2) It can be used as a method to evaluate the relative quality of the PacBio sequencing data by assembling a small batch of reads.

## Conclusions

Organelle_PBA is a program designed to assemble organelle genomes from PacBio whole genome sequencing data. Pre-selection of the organelle DNA sequencing reads using a mapping approach facilitates the organelle genome assembly and optimizes the computational requirements. It also removes the assembly redundancy caused by a circular assembly and resolves the chloroplast genome inverted repeats. Organelle_PBA performed successful assemblies of the mitochondrial and chloroplast genomes in model species such as *M. musculus* and *A. thaliana* respectively. The program was also used for the successful assembly of two novel chloroplast genomes from the species *Picea glauca* (a gymnosperm) and *Sinningia speciosa* (a eudicot angiosperm). The tool is freely available at https://github.com/aubombarely/Organelle_PBA.

## Availability and requirements

- Project name: Organelle_PBA
- Project home page: https://github.com/aubombarely/Organelle_PBA
- Archived version: Not applicable
- Operating system: Linux (tested on Ubuntu 14.04.1)
- Programming language: Perl
- Other requirements: Perl (5.18.2), BioPerl (1.6.924), Seqtk (1.0-r31), BlastN (2.3.0+), BlasR (1.3.1), Samtools (1.3-8-g03a6bc5), Bedtools (v2.17.0), Sprai (0.9.9.8), WGS-Assembler (8.3rc1), SSPACE-Long (v1-1).
- License: GNU General Public License

## Additional file

Additional file 1: Figure S1. Chart flow for the Organelle_PBA software. Reads are mapped to an organelle reference using BlasR (1). The BlasR output is parsed and the sequence IDs are used to retrieve the reads from the input file (2). Organelle identified reads are assembled using Sprai (3). The program checks if the assembly is complete comparing its length with the reference (4). If it is not complete, it performs a scaffolding using SSPACE-Long and the whole PacBio dataset (5). It is complete it moves to the new checking point where it check for circularity (6). If it detects circularity by a self-BlastN, it trims the sequence corresponding to the circular overlap (6). Finally it check for the repeat assembly (7) and if it finds it, it breaks in four parts, identify the complete inverted repeat (IR), duplicate it (IRa and IRb) and re-assemble it will the long and short single copy (LSC, SSC) (9). (TIFF 5352 kb)

## Abbreviations

DBG: De Bruijn Graph
InDel: Insertion-deletion
IR: Inverted repeat
LSC: Long single copy
OLC: Overlap–layout–consensus
SMRT: Single molecule real time
SNP: Single nucleotide polymorphism
SSC: Short single copy
