# Supplementary figures and images for: Organelle_PBA, a pipeline for assembling chloroplast and mitochondrial genomes from PacBio DNA sequencing data
Source: BMC Genomics. 2017 Jan 7;18:49. doi: 10.1186/s12864-016-3412-9 (PMC5219736; doi:10.1186/s12864-016-3412-9)

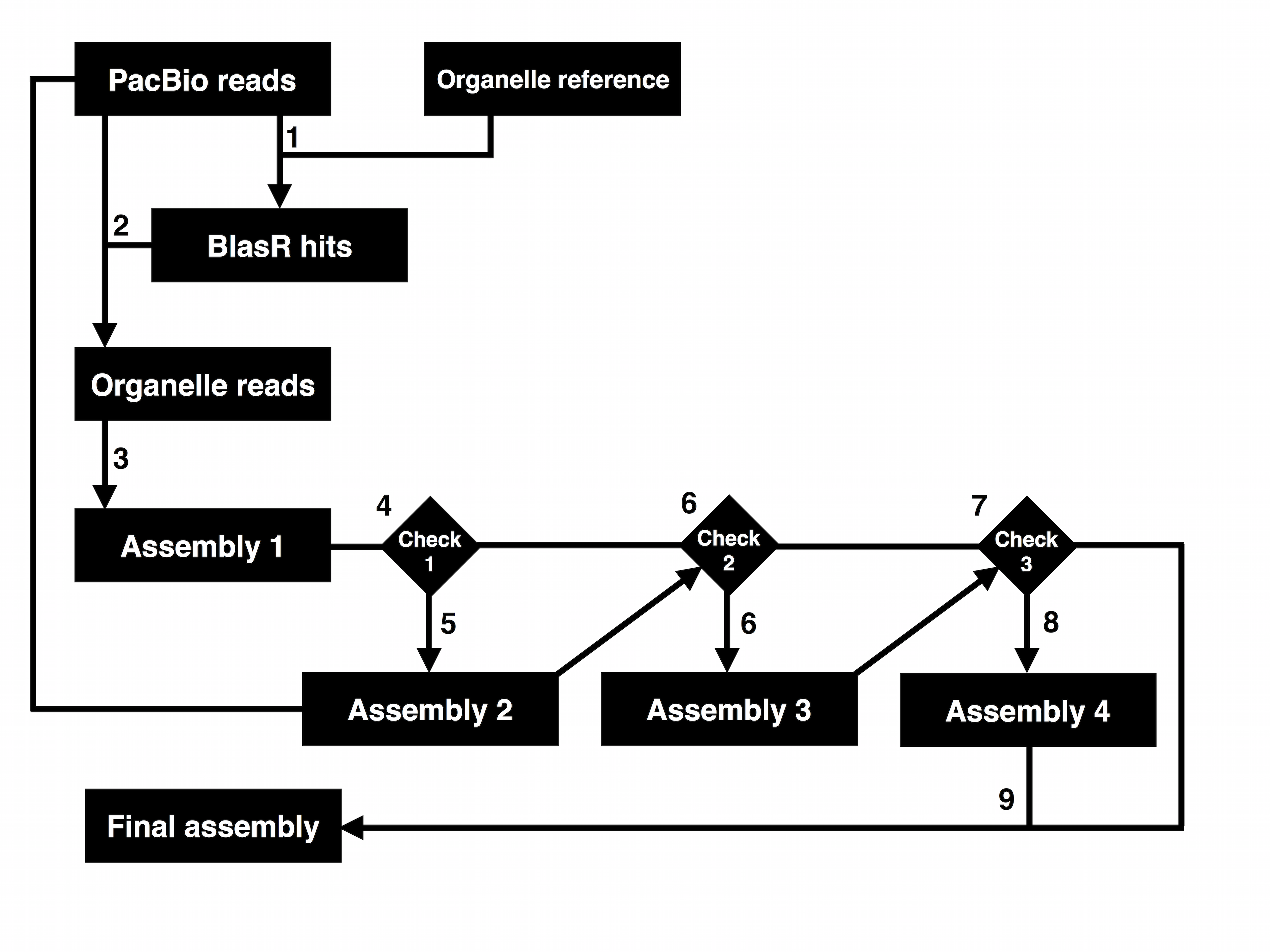

Supplement: Additional file 1: Figure S1. — Chart flow for the Organelle_PBA software. Reads are mapped to an organelle reference using BlasR (1). The BlasR output is parsed and the sequence IDs are used to retrieve the reads from the input file (2). Organelle identified reads are assembled using Sprai (3). The program checks if the assembly is complete comparing its length with the reference (4). If it is not complete, it performs a scaffolding using SSPACE-Long and the whole PacBio dataset (5). It is complete it moves to the new checking point where it check for circularity (6). If it detects circularity by a self-BlastN, it trims the sequence corresponding to the circular overlap (6). Finally it check for the repeat assembly (7) and if it finds it, it breaks in four parts, identify the complete inverted repeat (IR), duplicate it (IRa and IRb) and re-assemble it will the long and short single copy (LSC, SSC) (9). (TIFF 5352 kb) [file 12864_2016_3412_MOESM1_ESM.tiff]
